# Managing uterine evacuation in a patient with Asherman syndrome who failed procedural and medication abortions: a case report

**DOI:** 10.1016/j.crwh.2026.e00856

**Published:** 2026-09-09

**Authors:** Morgan E. Johnson, Jaime A. Roura-Monllor, Adam D. Elwood, Anne Z. Steiner, Jessica E. Morse

**Affiliations:** a University of North Carolina at Chapel Hill School of Medicine, Chapel Hill, North Carolina; b Department of Obstetrics and Gynecology, Reproductive Endocrinology & Infertility Division, University of North Carolina at Chapel Hill, Chapel Hill, North Carolina; c Department of Obstetrics and Gynecology, Complex Family Planning Division, University of North Carolina at Chapel Hill, Chapel Hill, North Carolina

**Keywords:** Abortion, Intrauterine adhesions, Asherman syndrome, Failed abortion, Case report

## Abstract

This report describes the case of a 37-year-old woman (G5P1041) with Asherman syndrome who failed both medication and procedural abortions. Her medical history included recurrent pregnancy loss, postpartum hemorrhage, and multiple suction and sharp dilation and curettages. She was found to have intrauterine adhesions and while awaiting lysis of adhesions, she became pregnant and desired termination of pregnancy. The patient's clinical course was as follows: unsuccessful vacuum aspiration at 6 weeks 0 days due to inability to reach the gestational sac, failed medication abortion, unsuccessful hysteroscopic abortion with morcellation at 6 weeks 5 days, and successful hysteroscopic removal of retained products of conception one month later.

Clinical guidance is lacking for patients with intrauterine adhesions that fail procedural and medication abortions. This single case suggests that management modalities to consider include comprehensive preoperative planning, ultrasound guidance, hysteroscopic removal of the products of conception, use of second-look hysteroscopy, and close postoperative follow-up.

## Highlights

• A patient with Asherman syndrome failed both medication and procedural abortions.
• This patient's intrauterine adhesions may have contributed to failure.
• Ultrasound guidance and/or hysteroscopy may be potential management options.

## 1 Introduction

Asherman syndrome refers to intrauterine adhesions with symptoms such as pelvic pain, menstrual disturbance, recurrent pregnancy loss, or infertility [1]. The severity of intrauterine adhesions can range from diffuse, thin, and filmy to thick and fibrous bands to effective obliteration of the entire endometrial cavity [2]. Well-documented sequalae include infertility, recurrent pregnancy loss, and obstetric complications such as preterm birth, abnormal placentation, placental insufficiency, and peripartum hemorrhage [3], [4], [5], [6]. Endometrial instrumentation causing injury to the decidua basalis is understood to lead to this syndrome [2], [7].

While the etiology, clinical presentation, and sequelae of Asherman syndrome are largely well defined, the literature does not describe whether these patients are at an increased risk of failing medication and/or procedural abortion. Failure is defined as retained products of conception. It is theoretically plausible that intrauterine adhesions could increase the risk of failing medication and/or procedural abortion(s) due to products of conception being retained behind adhesions. Both medication and procedural abortions are considered safe and effective [8], with failure rates ranging from 0.3 to 8.4% for medication abortion [[9], [17]] and from 0 to 2.5% for procedural abortions [10], [11], [12], [13]. There are no documented risk factors for failing both medication and procedural abortions, likely due to how rarely dual failure occurs.

This report describes a patient with Asherman syndrome who became pregnant prior to planned adhesiolysis and subsequently failed medication and procedural abortions. The literature is briefly reviewed on the approach to uterine evacuation in patients with Asherman syndrome. Gaps in the literature are highlighted, with the aim of inspiring evidence-based management strategies to best care for these patients.

## 2 Case Presentation

A 37-year-old G5P1041 woman sought evaluation with the reproductive endocrinology and infertility department for recurrent pregnancy loss. Her first pregnancy resulted in a spontaneous miscarriage at five weeks. Her second pregnancy was a term, spontaneous vaginal delivery complicated by delayed postpartum hemorrhage requiring blood transfusion and combined suction and sharp uterine curettage. Her third and fourth pregnancies were biochemical pregnancy losses. Her fifth pregnancy resulted in a 7–8-week early pregnancy loss managed with suction and sharp D&C followed by a repeat suction D&C for retained products of conception two months later.

**Fig. 1:**
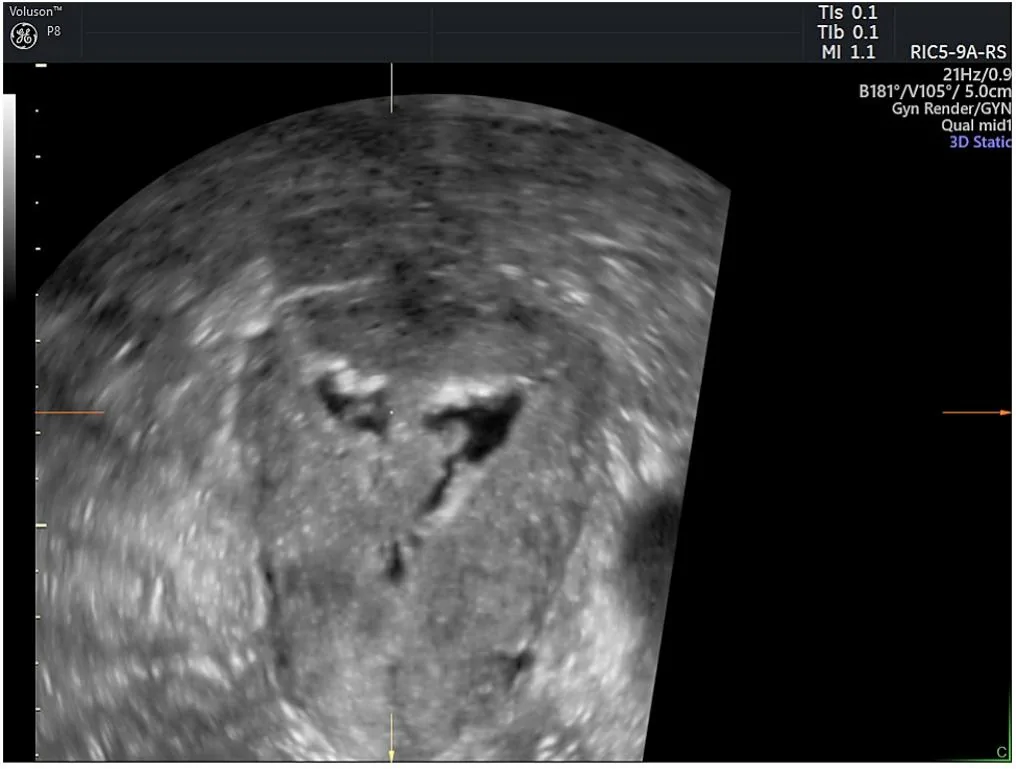
Saline sonohysterogram showing a large intrauterine filling defect consistent with intrauterine adhesions.

During her evaluation for recurrent pregnancy loss, she underwent saline infusion hysterography, which was remarkable for a large intrauterine filling defect consistent with intrauterine adhesions (Fig. 1). She was scheduled for hysteroscopic adhesiolysis (Fig. 1). While awaiting this procedure, she became pregnant without medical assistance. She was concerned that this pregnancy would lead to another loss and/or other pregnancy complications, so she opted for termination of pregnancy.

She presented to an ambulatory clinic at 6 weeks 0 days of gestation for a scheduled ultrasound-guided uterine vacuum aspiration. Following cervical dilation, a suction cannula was passed through the cervical os. The gestational sac was unable to be reached with the suction cannula due to the breadth and depth of adhesive disease and the attempt was aborted. Given this unsuccessful procedure and early gestational age, she proceeded with a medication abortion. She received mifepristone 200 mg orally that day and misoprostol 800 μg to be placed buccally the following day in the standard fashion. Hours after placing the misoprostol buccally, at 6 weeks 1 day, the patient reported no bleeding or cramping. A second dose of misoprostol 800 μg buccally was recommended and self-administered. Two days later, she presented to the family planning clinic with minimal vaginal bleeding and cramping. A repeat transvaginal ultrasound confirmed an intrauterine pregnancy.

**Fig. 2:**
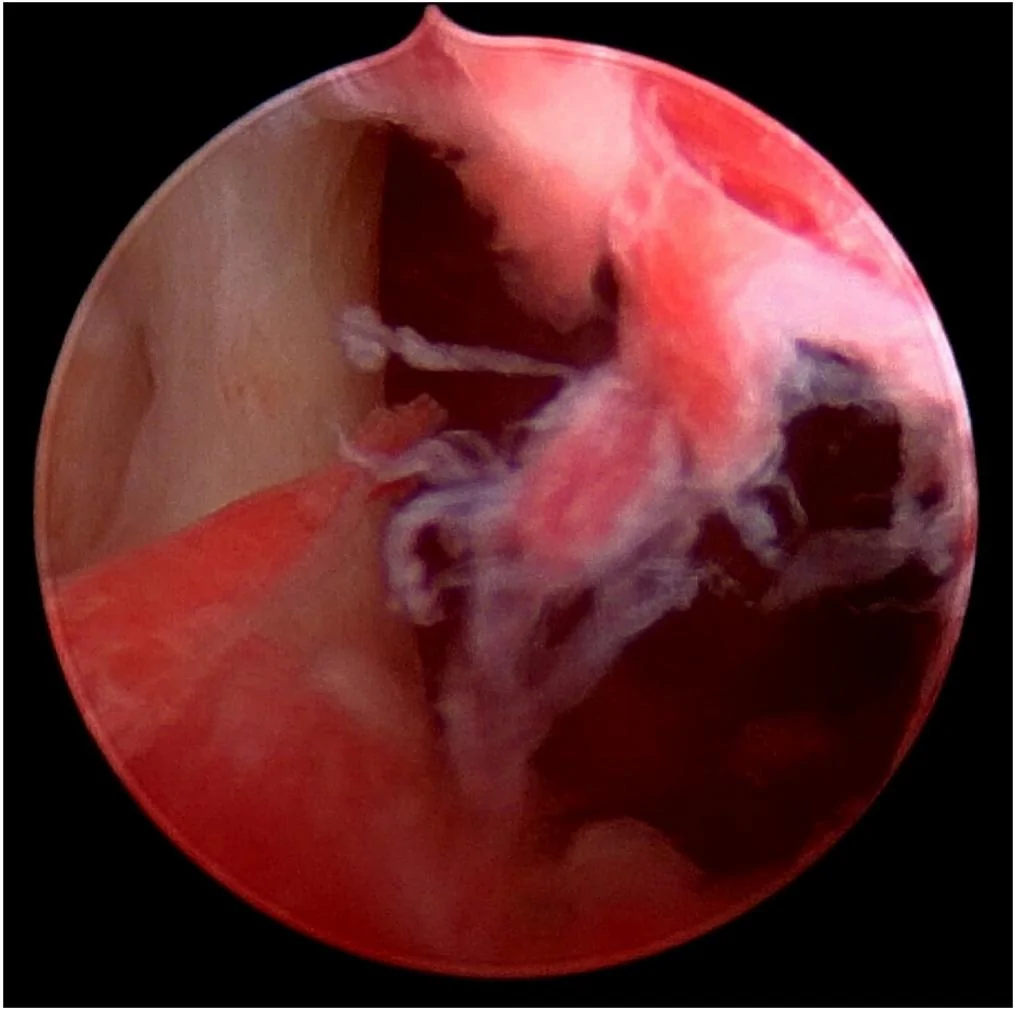

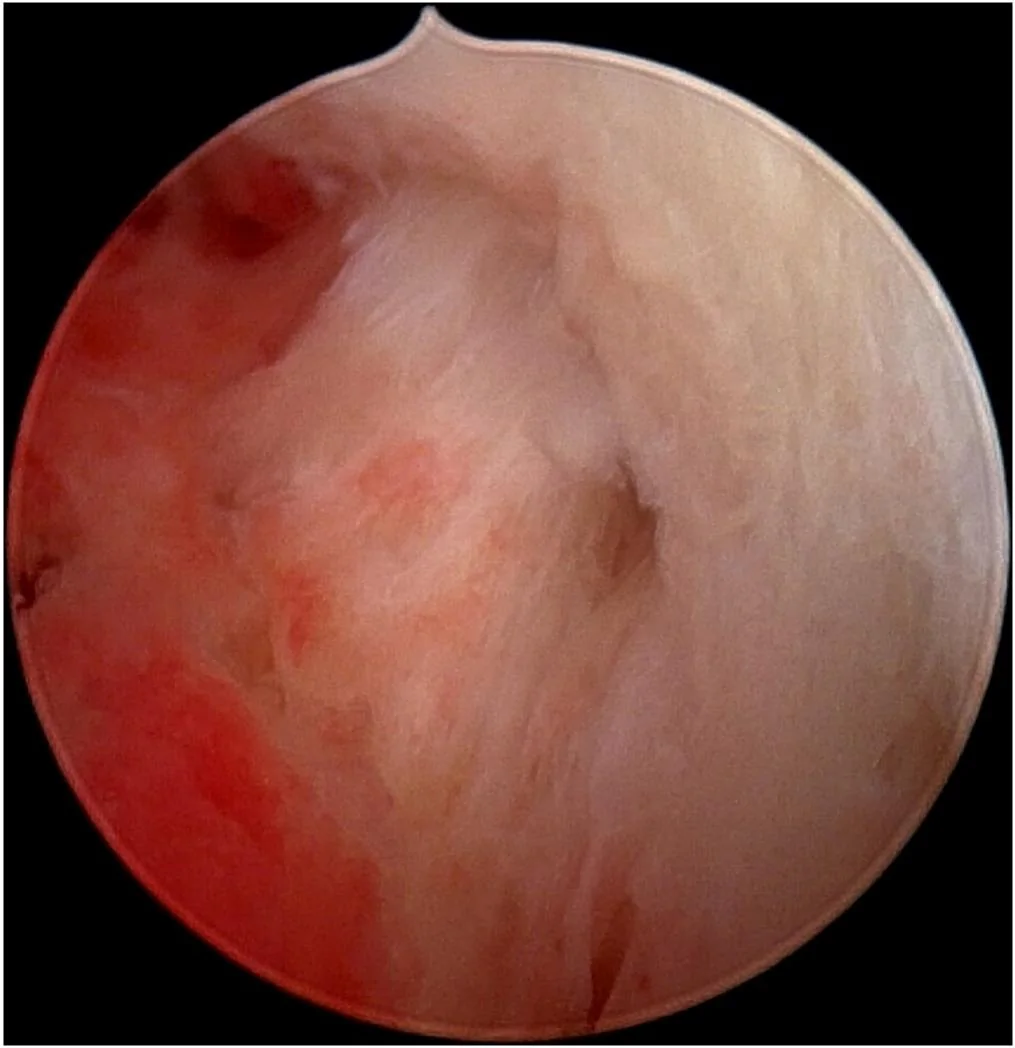
Hysteroscopy showing (a) a thick anteroposterior mid-to-lower cavity intrauterine adhesion and (b) an intrauterine pregnancy in the left upper fundus.

At 6 weeks 5 days, she underwent hysteroscopy. Hysteroscopy revealed dense intrauterine adhesions dividing the uterine cavity into two sections (Fig. 2a). Products of conception were seen in the left cornua (Fig. 2b) behind thick adhesions and confirmed with transabdominal ultrasound. Adhesions were lysed sharply with scissors, and the tissue was removed with a hysteroscopic morcellator. At the end of the procedure, the cavity appeared normal. The left tubal ostia could not be visualized. Ultrasound showed a normal endometrial echo complex suggesting successful removal of products of conception. An eight french balloon catheter was inserted into the uterine cavity and filled with 3 mL of saline with planned removal 1 week postoperatively. The patient was discharged with doxycycline 100 mg oral twice daily for 1 week for infection prophylaxis, estradiol 2 mg oral daily for 1 month to decrease risk of adhesion recurrence, and medroxyprogesterone acetate 10 mg oral daily for the last 10 days of estradiol therapy to induce menses. Multimodal postoperative pain management included acetaminophen, ibuprofen, and opioids. Surgical pathology showed immature hydropic chorionic villi without fetal tissue and without evidence of gestational trophoblastic disease.

The patient's postoperative course was remarkable for acute severe pain associated with vaginal bleeding that prompted emergency department visits on postoperative days 1 and 5 where the intrauterine balloon catheter was noted to be in situ. The patient was discharged from the emergency department with a multimodal pain regimen and underwent uncomplicated catheter removal in the office on postoperative day 6.

**Fig. 3:**
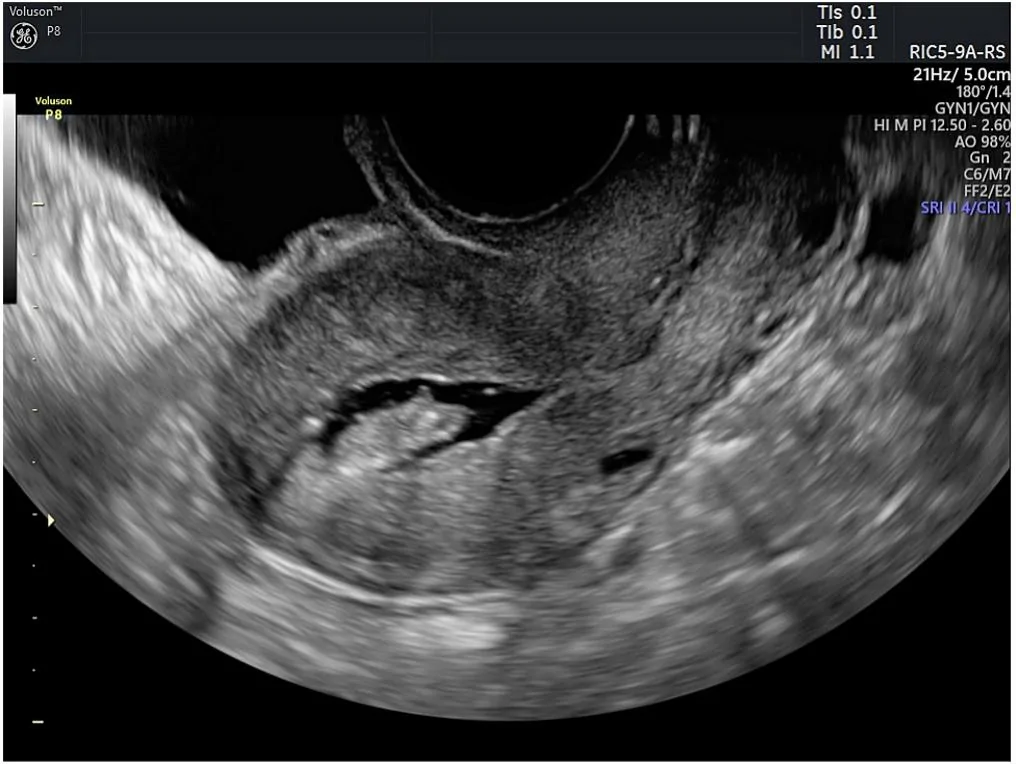

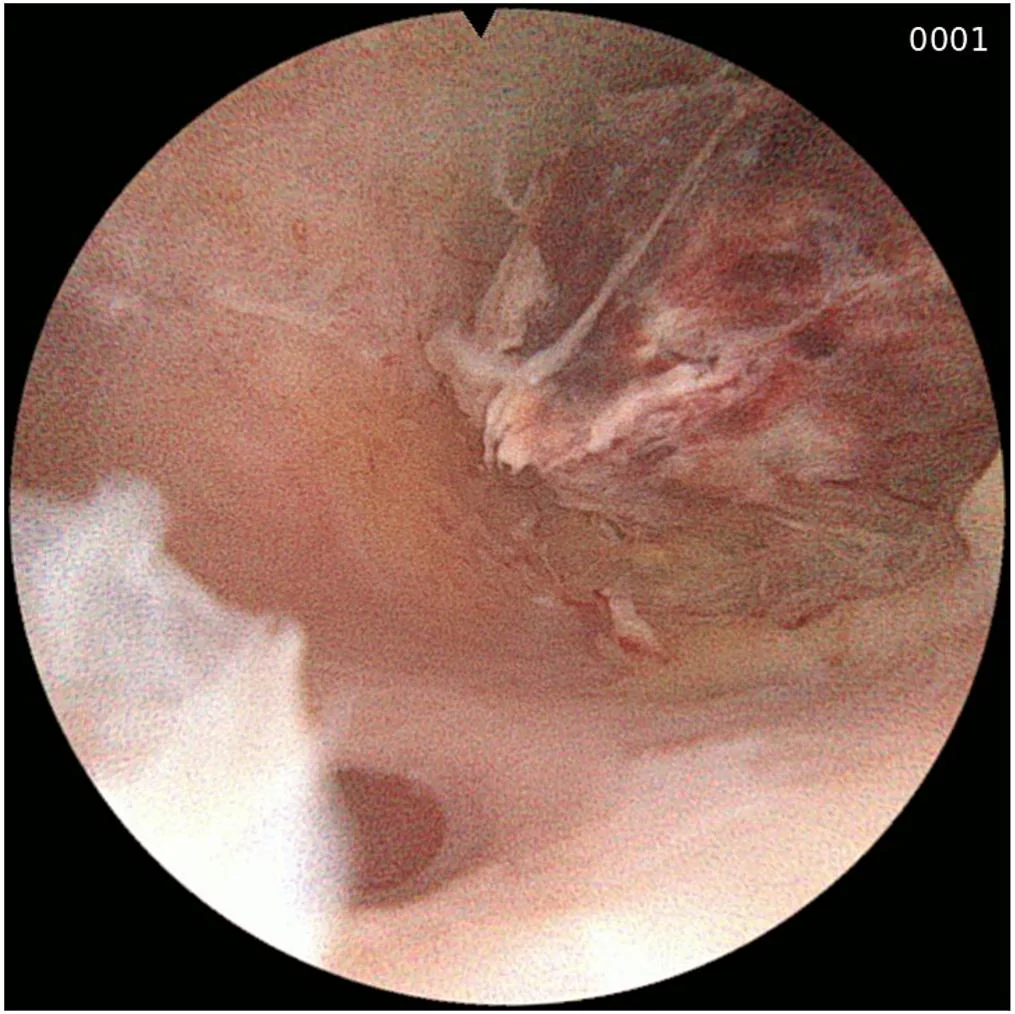

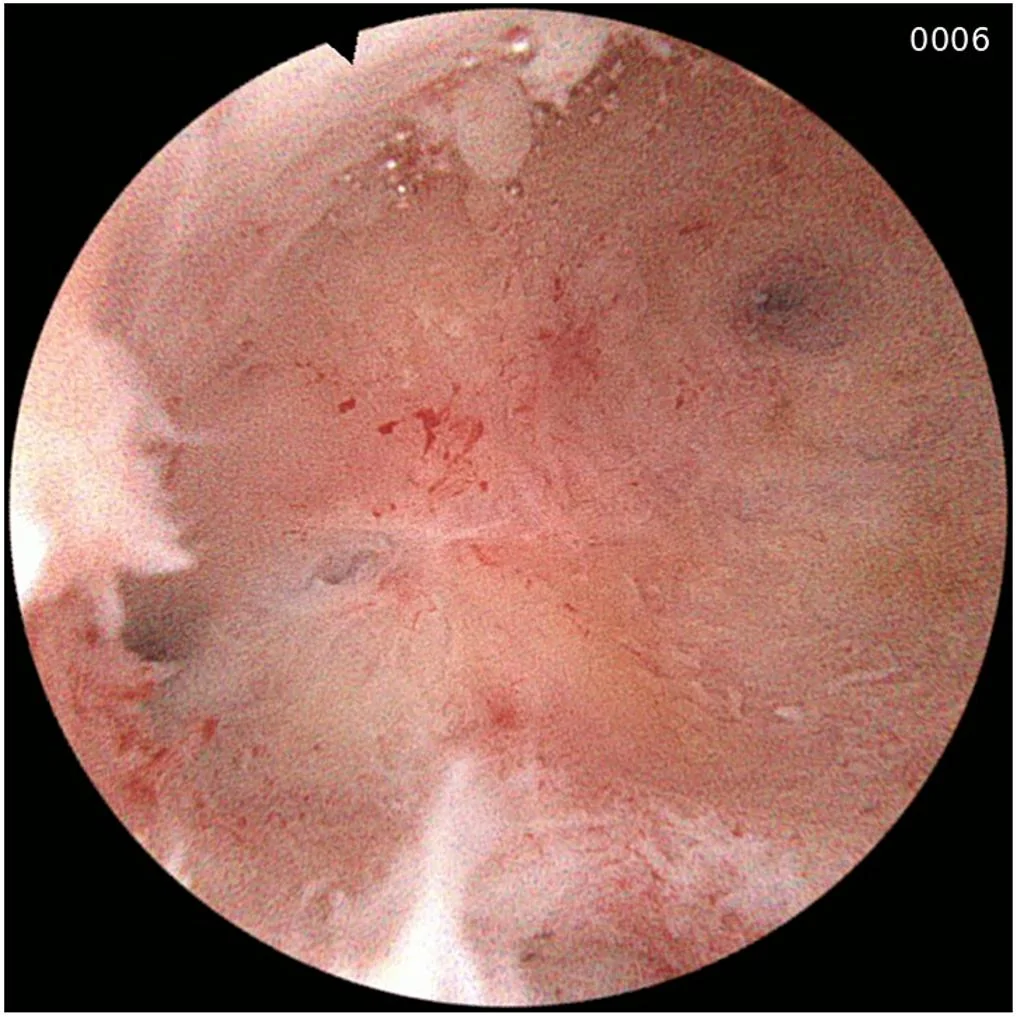
Follow-up (a) saline sonohysterogram and (b) hysteroscopy showing tissue consistent with products of conception. Note an oblique posterior wall adhesion at the upper cavity in (b). Panel (c) shows the cavity and ostia at the end of the procedure.

Saline sonohysterography conducted one month post-operatively following the withdrawal bleed from medroxyprogesterone acetate showed an intrauterine filling defect concerning for retained products of conception (Fig. 3a). She subsequently underwent hysteroscopy and a hysteroscopic morcellator was used to remove the retained products of conception. Pathology showed fibrotic chorionic villi in a background of decidua and hemorrhage, consistent with retained products of conception.

At follow-up, she reported a biochemical pregnancy loss. She underwent saline sonohysterography and hysteroscopy for further uterine evaluation. Suboptimal distension and a fundal filling defect were noted on saline sonohysterography. Hysteroscopy showed normal ostia and minimal fundal scarring that was lysed.

**Fig. 4:**
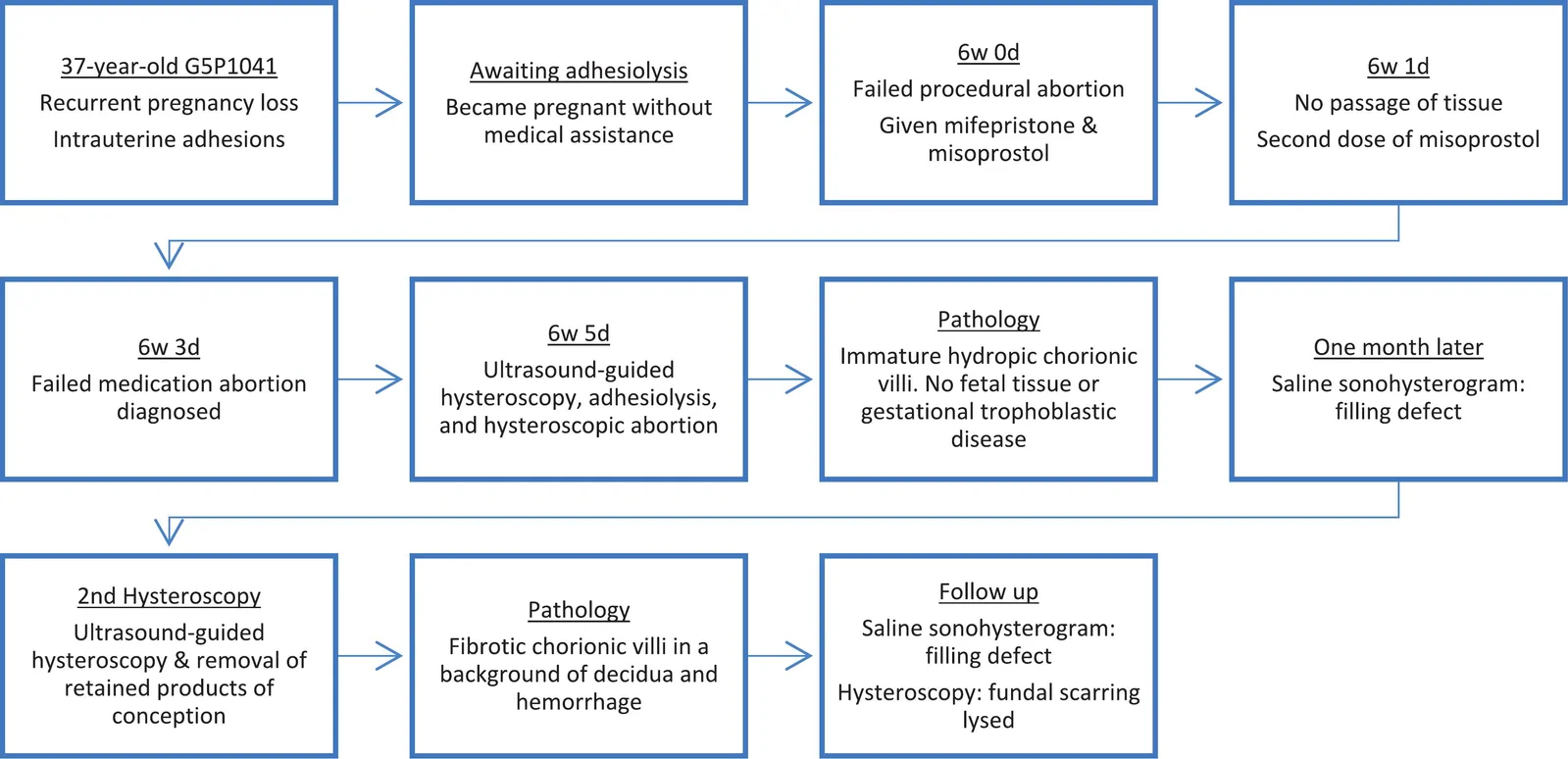
Timeline of events.

The timeline of the case is summarized in Fig. 4.

At the time of writing, the patient was planning in vitro fertilization with pre-implantation genetic testing for aneuploidy.

## 3 Discussion

This report describes a patient with a history of suction D&Cs for a miscarriage and for retained products of conception, one postpartum curettage, known intrauterine adhesions, and recurrent pregnancy loss who presented for pregnancy termination and subsequently failed procedural and medication abortions. Given the lack of guidance for failed procedural and medication abortion in patients with intrauterine adhesions, it is important to contextualize this case relative to the etiology of intrauterine adhesions and other case reports.

This patient's initial vacuum aspiration was technically unsuccessful given the inability to bypass dense intrauterine adhesions. It is plausible that her subsequent failed medication and procedural abortions were due to intrauterine adhesions. Given the rarity of abortion failure, clinicians should be aware of other possible etiologies of failed abortion. Other risk factors include improper technique, cervical stenosis, endometrial distortion, uterine fibroids, and uterine anomaly [14]. Magnetic resonance imaging may be helpful to evaluate for uterine fibroids or anomaly when suspected.

There are case reports that describe failed abortions in the setting of intrauterine adhesions. One detailed a patient who failed three suction D&Cs, underwent exploratory laparotomy for suspected ectopic pregnancy, failed methotrexate therapy, and then hysteroscopy, where access to products of conception was limited by dense adhesions [15]. During hysteroscopy, the pregnancy was evacuated using ultrasound-guided blunt lysis of intrauterine adhesions and suction D&C. Another report described failed misoprostol treatment in the setting of Asherman syndrome requiring hysteroscopic adhesiolysis and subsequent removal of the retained products of conception [16].

Currently there are no published guidelines on failed abortions in the setting of intrauterine adhesions. General recommendations for failed medication abortion include repeating misoprostol, uterine aspiration, or expectant management with counseling about possible adverse events [9]. General recommendations for failed procedural abortions are to repeat prior procedural abortion type (i.e., vacuum aspiration or D&C) or attempt medication abortion for patients with altered anatomy who have not already failed medication abortion [12]. Ultrasound guidance may increase the likelihood of successful abortion in cases of uterine anomalies. Similarly, no studies have evaluated the management of miscarriage in patients with Asherman syndrome.

Providers may consider using ultrasound guidance during procedural abortions and hysteroscopic management. The literature indicates that operative hysteroscopy in the setting of retained products of conception is safe and effective and associated with decreased risks of post-operative adhesions [18]. Of note, in this case, the first attempted hysteroscopic lysis of adhesions and abortion was unsuccessful. It is possible that dense adhesive tissue obscured some of the pregnancy tissue. Ultimately, repeat ultrasound-guided hysteroscopy successfully removed retained products. This reinforces that future research should evaluate management modalities and that adhesion-related difficulties may complicate the clinical course.

This and other case reports suggest the possibility that patients with adhesions may be at risk for failing standard medical and surgical methods for pregnancy evacuation. Future studies should quantify the risk of failed medical and surgical evacuation of pregnancy in patients with Asherman and create criteria for using standard pregnancy evacuation techniques versus hysteroscopic approaches. While the optimal management approach is unclear, these patients should have close follow-up and be engaged in shared decision-making.

## Contributors

Morgan E. Johnson contributed to conception of the case report, drafting the manuscript, undertaking the literature review and revising the article critically for important intellectual content.

Jaime A. Roura-Monllor contributed to patient care, conception of the case report, acquiring and interpreting the data and revising the article critically for important intellectual content.

Adam D. Elwood contributed to patient care, conception of the case report, acquiring and interpreting the data and revising the article critically for important intellectual content.

Anne Z. Steiner contributed to patient care and revising the article critically for important intellectual content.

Jessica E. Morse contributed to patient care and revising the article critically for important intellectual content.

All authors approved the final submitted manuscript.

## Patient consent

Written informed consent was obtained from the patient for publication of this case report and accompanying images.

## Provenance and peer review

This article was not commissioned and was peer reviewed.

## Ethics declaration

Written informed consent to take part in the study and to publish the article has been obtained from all participants or their legal representatives. The privacy rights of participants have been observed.

This study was performed in compliance with relevant laws, regulatory frameworks and guidelines where the research took place.

Ethics committee approval was not required under relevant laws and institutional guidelines. As per institutional policy, single patient case reports do not require Institutional Review Board (IRB) review.

This research follows the CARE guidelines and the CARE checklist.

## Declaration of generative AI and AI-assisted technologies in the writing process

Jaime Roura-Monllor utilized artificial intelligence to address writer's block. Targeted prompts were employed to clarify intended messages, ensuring that all resulting content was original.

## Funding

This work did not receive any specific grant from funding agencies in the public, commercial, or not-for-profit sectors.

## Declaration of competing interest

Morgan Johnson has nothing to disclose.

Jaime Roura-Monllor is a paid peer reviewer for Doximity, unrelated to this case report.

Adam Elwood has nothing to disclose.

Anne Steiner currently receives funding from May Health for research and consulting fees from SPD (Clearblue), unrelated to this case report.

Jessica Morse has nothing to disclose.
